# An ant colony optimisation algorithm for the 2D and 3D hydrophobic polar protein folding problem

**DOI:** 10.1186/1471-2105-6-30

**Published:** 2005-02-14

**Authors:** Alena Shmygelska, Holger H Hoos

**Affiliations:** 1 Department of Computer Science, University of British Columbia, Vancouver, B.C., V6T 1Z4, Canada

## Abstract

**Background:**

The protein folding problem is a fundamental problems in computational molecular biology and biochemical physics. Various optimisation methods have been applied to formulations of the *ab-initio *folding problem that are based on reduced models of protein structure, including Monte Carlo methods, Evolutionary Algorithms, Tabu Search and hybrid approaches. In our work, we have introduced an ant colony optimisation (ACO) algorithm to address the non-deterministic polynomial-time hard (*NP*-hard) combinatorial problem of predicting a protein's conformation from its amino acid sequence under a widely studied, conceptually simple model – the 2-dimensional (2D) and 3-dimensional (3D) hydrophobic-polar (HP) model.

**Results:**

We present an improvement of our previous ACO algorithm for the 2D HP model and its extension to the 3D HP model. We show that this new algorithm, dubbed ACO-HPPFP-3, performs better than previous state-of-the-art algorithms on sequences whose native conformations do not contain structural nuclei (parts of the native fold that predominantly consist of local interactions) at the ends, but rather in the middle of the sequence, and that it generally finds a more diverse set of native conformations.

**Conclusions:**

The application of ACO to this bioinformatics problem compares favourably with specialised, state-of-the-art methods for the 2D and 3D HP protein folding problem; our empirical results indicate that our rather simple ACO algorithm scales worse with sequence length but usually finds a more diverse ensemble of native states. Therefore the development of ACO algorithms for more complex and realistic models of protein structure holds significant promise.

## Background

Ant Colony Optimisation (ACO) is a population-based stochastic search method for solving a wide range of combinatorial optimisation problems. ACO is based on the concept of *stigmergy *– indirect communication between members of a population through interaction with the environment. An example of stigmergy is the communication of ants during the foraging process: ants indirectly communicate with each other by depositing pheromone trails on the ground and thereby influencing the decision processes of other ants. This simple form of communication between individual ants gives rise to complex behaviours and capabilities of the colony as a whole.

From the computational point of view, ACO is an iterative construction search method in which a population of simple agents ('ants') repeatedly constructs candidate solutions to a given problem; this construction process is probabilistically guided by heuristic information on the given problem instance as well as by a shared memory containing experience gathered by the ants in previous iterations ('pheromone trails'). Following the seminal work by Dorigo *et al. *[1,2], ACO algorithms have been successfully applied to a broad range of hard combinatorial problems, including the traveling salesman problem, the graph colouring problem, the quadratic assignment problem and vehicle routing problems (see, *e.g., *[3-5]).

The research presented in this paper builds on an ACO algorithm first proposed in [6] (and later improved in [7]) for *ab-initio *protein folding under a widely studied abstract model – the hydrophobic polar (HP) model. In particular, we extend our previous ACO algorithm to the 3D HP model and improve its performance by modifying the subsidiary local search procedure.

The protein folding problem is one of the most challenging problems in computational biology, molecular biology, biochemistry and physics. Even under simplified lattice models, the protein folding problem is non-deterministic polynomial-time hard (*NP*-hard) [8]. The *ab-initio *protein folding problem can be broken down into three sub-problems: 1) design of a model (with a desired level of accuracy); 2) definition of an energy function that can effectively discriminate between native and non-native states; and 3) design of a search algorithm that can efficiently find minimal-energy conformations. A number of search (or sampling) methods have been proposed in the literature to solve the protein folding problem, including Monte Carlo algorithms, Evolutionary Algorithms, Tabu Search and hybrid approaches. ACO, which has been very successfully applied to other combinatorial problems, appears to be a very attractive computational method for solving the protein folding problem, since it combines aspects of chain-growth and permutation-based search with ideas closely related to reinforcement learning. These concepts and ideas apply rather naturally to protein folding: By folding from multiple initial folding points, guided by the energy function and experience from previous iterations of the algorithm, an ensemble of promising, low-energy complete conformations is obtained. These conformations are further improved by a subsidiary local search procedure and then evaluated to update the accumulated pheromone values that are used to bias the generation of conformations in future iterations of the algorithm.

In this paper, we ask and address the following questions: Is ACO a competitive method for solving the *ab-initio *protein folding problem under the 2D and 3D HP models? How does its performance scale with sequence length? What is the role of the parameters of the ACO algorithm for the efficiency of the optimisation process? Which classes of structures (if any) are solved more efficiently by ACO than by any other known algorithms? Finally, it should be noted that our ACO algorithm for this problem is based on very simple design choices, in particular with respect to the solution components reinforced in the pheromone matrix and of the subsidiary local search procedure. We discuss which of the many design choices underlying our algorithm should be reconsidered in order to achieve further performance improvements.

### The hydrophobic polar model

Due to the complexity of the protein folding problem, simplified models such as Dill's hydrophobic-polar (HP) model have become one of the major tools for studying protein structure [9]. The HP model is based on the observation that the hydrophobic force is the main force determining the unique native conformation (and hence the functional state) of small globular proteins [9,10].

**Figure 1:**
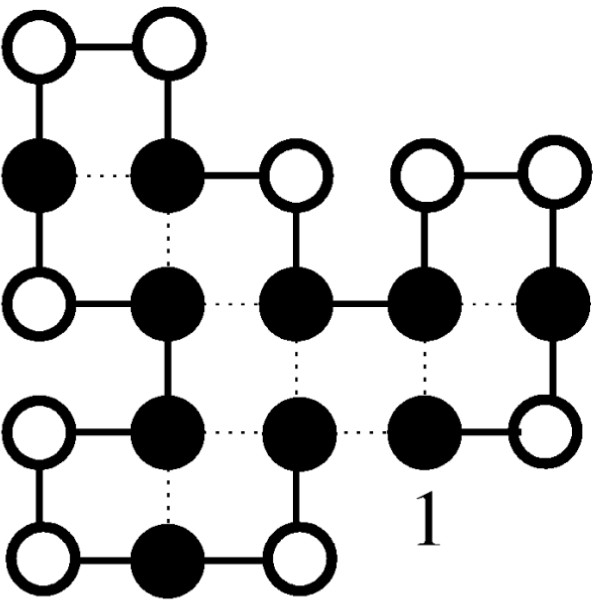
**A sample protein conformation in the 2D HP model**. The underlying protein sequence (Sequence S1-1 from Table 1)is HPHPPHHPHPPHPHHPPHPH; black circles represent hydrophobic amino acids, while white circles symbolise polar amino acids. The dotted lines represents the H-H contacts underlying the energy calculation. The energy of this conformation is -9, which is optimal for the given sequence.

In the HP model, the primary amino acid sequence of a protein (which can be represented as a string over a twenty-letter alphabet) is abstracted to a sequence of hydrophobic (H) and polar (P) residues that is represented as a string over the letters H and P. The conformations of such an HP sequence are restricted to self-avoiding walks on a lattice. For the 2D HP model, a 2-dimensional square lattice is typically used, and the 3D HP model is generally based on a 3-dimensional cubic lattice. An example of a protein conformation under the 2D HP model is shown in Figure 1. The energy of a conformation is defined as the number of topological contacts between hydrophobic amino acids that are not neighbours in the given sequence. More specifically, a conformation *c *with exactly *n *such H-H contacts has energy *E*(*c*) = *n*·(-1); for example, the 2D HP conformation shown in Figure 1 has energy -9.

The HP Protein Folding Problem can be formally defined as follows: Given an HP sequence *s *= *s*_1 _*s*_2_...*s*_*n*_, find an energy-minimising conformation of *s*, *i.e., *find c* ∈ *C*(*s*) such that *E*(c*) = min{*E*(*c*) | *c *∈ *C*}, where *C*(*s*) is the set of all valid conformations for *s*. It has been proved recently that this problem and several variations of it are *NP*-hard [8].

### Existing 2D and 3D HP protein folding algorithms

A number of well-known heuristic optimisation methods have been applied to the 2D and 3D HP Protein Folding Problem, including Evolutionary Algorithms (EAs) [11-15] and Monte Carlo (MC) algorithms [16-22]. The latter have been found to be particularly robust and effective for finding high-quality solutions to the HP Protein Folding Problem [18].

Besides general optimisation methods, there are other heuristic methods that rely on specific heuristics that are based on intuitions or assumptions about the folding process, such as co-operativity of folding or the existence of a hydrophobic core. Co-operativity is believed to arise from local conformational choices that result in a globally optimal state without exhaustive search [23]. Among these methods are the hydrophobic zipper method (HZ) [23], the contact interactions method (CI) [24], the core-directed chain growth method (CG) [25], and the constraint-based hydrophobic core construction method (CHCC) [26].

The hydrophobic zipper (HZ) strategy developed by Dill *et al. *is based on the hypothesis that once a hydrophobic contact is formed it cannot be broken, and other contacts are formed in accordance with already folded parts of the chain (co-operativity of folding) [23]. The contact interactions (CI) algorithm by Toma and Toma [24] combines the idea of HZ with a Monte Carlo search procedure that assigns different conformational freedom to the different residues in the chain, and thus allows previously formed contacts to be modified according to their computed mobilities. The core-directed chain growth method (CG) by Beutler and Dill [25] biases construction towards finding a good hydrophobic core by using a specifically designed heuristic function and by approximating the hydrophobic core with a square (in 2D) or a cube (in 3D). The constraint-based hydrophobic core construction method (CHCC) by Yue and Dill [26] is complete, *i.e., *always guaranteed to find a global optimum; it attempts to find the hydrophobic core with the minimal possible surface area by systematically introducing geometric constraints and by pruning branches of a conformational search tree. A similar, but more efficient complete constraint satisfaction search method has been proposed by Backofen *et al. *[27] for the more complex face-centred cubic lattice.

An early application of Evolutionary Algorithms to protein structure prediction was presented by Unger and Moult [14,15]. Their non-standard EA incorporates characteristics of Monte Carlo methods. Currently among the best known algorithms for the HP Protein Folding problem are various Monte Carlo algorithms, including the 'pruned-enriched Rosenbluth method' (PERM) of Grassberger *et al. *[16,18]. PERM is a biased chain growth algorithm that evaluates partial conformations and employs pruning and enrichment strategies to explore promising partial solutions.

Other methods for solving protein folding problems include the dynamic Monte Carlo algorithm by Ramakrishnan *et al. *[21], which introduced long-range moves involving disconnection of the chain, and the evolutionary Monte Carlo (EMC) algorithm by Liang and Wong [19], which works with a population of individuals that each perform Monte Carlo optimisation; a variant of EMC also reinforces certain secondary structures (alpha-helices and beta-sheets).

Finally, Chikenji *et al. *introduced the multi-self-overlap ensemble (MSOE) Monte Carlo method [17], which considers overlapping chain configurations.

Other Monte Carlo methods that have been particularly useful in off-lattice protein folding include generalised ensemble methods, such as umbrella sampling [28] (with replica exchange sampling [29,30] being the most common variant) and multi-canonical (entropic) sampling [30,31]. Replica exchange Monte Carlo (parallel tempering) has also been applied to the off-lattice HP model [32].

Currently, when applied to the square and cubic lattice HP model, none of these algorithms appears to completely dominate the others in terms of solution quality and run-time.

### Our ACO algorithm for the 2D and 3D HP protein folding problem

In previous work, we have applied ACO to the 2D HP Protein Folding Problem [6,7]; in the following, we briefly summarise the main features of our ACO algorithm and the improvements introduced in this work. Details on our ACO framework and the new ACO-HPPFP-3 algorithm developed in the context of this work are given in the 'Methods' section.

**Figure 2:**
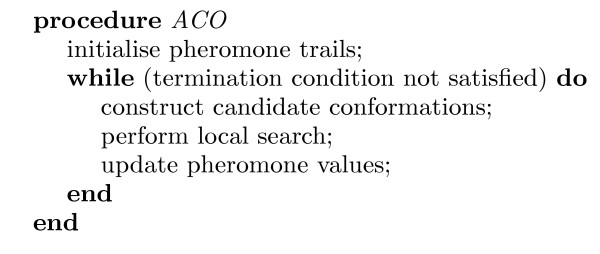
**ACO outline**. Generic outline of Ant Colony Optimisation (for static combinatorial problems).

As usual, the ants in our ACO algorithm iteratively undergo three phases: the *construction phase *– during which each ant constructs a candidate solution by sequentially growing a conformation of the given HP sequence, starting from a folding point that is chosen uniformly at random among all sequence positions; the *local search phase *– when ants further optimise protein conformations folded during the construction phase; and the *pheromone update phase *– when ants update the pheromone matrix (representing the collective global memory of the colony) based on the energies of the conformations obtained after the construction and the local search phases. A general outline of ACO is shown in Figure 2.

**Figure 3:**
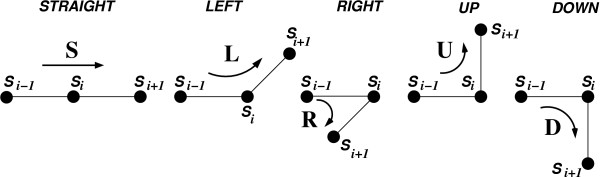
**Local structure motifs**. The local structure motifs which form the solution components underlying the construction and local search phases of our ACO algorithm in 3D.

The solution components used during the construction process, the local search phase and the pheromone update are local structure motifs (or relative folding directions) *straight *(S), *left *(L), *right *(R) in 2D, and *straight *(S), *left *(L), *right *(R), *up *(U), *down *(D) in 3D, which for each amino acid indicate its position on the 2D or 3D lattice relative to its direct predecessors in the given sequence (see Figure 3). In 3D, the relative folding directions are defined as in [33]: A local coordinate system is associated with every sequence position, such that *S *corresponds to the direction of the *x *axis, *L *to the direction of the *y *axis, and *U *to the direction of the *z *axis. Each local motif corresponds to a relative rotation of this coordinate system (for the forward construction: S = no rotation, L = 90° counter-clockwise around the *z *axis, R = 90° clockwise around the *z *axis, U = 90° clockwise around the *y *axis, D = 90° counter-clockwise around the *y *axis).

Since conformations are rotationally invariant, the position of the first two amino acids can be fixed without loss of generality. Hence, we represent candidate conformations for a protein sequence of length *n *by a sequence of local structure motifs of length *n *- 2. For example, the conformation of Sequence S1-1 shown in Figure 1 corresponds to the motif sequence LSLLRRLRLLSLRRLLSL.

During the construction phase, ants fold a protein from an initial folding point by probabilistically adding one amino acid at a time based on the two sources of information: pheromone matrix values *τ *(which represent previous search experience and reinforce certain structural motifs) and heuristic function values *η *(which reflect current energy of the considered structural motif); details of this process are given in the 'Methods' section. The relative importance of *τ *and *η *is determined by parameters *α *and *β*, respectively, whose settings are detailed in the 'Discussion' section. Similar to other ACO algorithms known from the literature, our algorithm for the HP Protein Folding Problem incorporates a local search phase that takes the initially built protein conformation and attempts to optimise its energy further, using probabilistic long-range moves that are described in detail in the 'Methods' section.

Finally, the pheromone update procedure is based on two mechanisms: Uniform pheromone evaporation is modelled by decreasing all pheromone levels by a constant factor *ρ *(where 0 <*ρ *≤ 1), and pheromone reinforcement is achieved by increasing the pheromone levels associated with the local folding motifs used in a fraction of the best conformations (in terms of energy values) obtained during the preceding construction and local search phase. Furthermore, to prevent search stagnation when all of the pheromone is accumulated on very few structural motifs, we introduce an additional renormalisation mechanism for the pheromone levels (controlled by a parameter *θ *where 0 ≤ *θ *< 1; details are given in the 'Methods' section).

Different from our previous ACO algorithms for the HP Protein Folding Problem, our new algorithm, ACO-HPPFP-3, supports the 3D HP cubic lattice model in addition to the 2D HP square lattice model. Furthermore, it uses a different iterative improvement strategy, a modified long-range move operator and a less restrictive termination criterion in its local search phase. ACO-HPPFP-3 was used in all ACO experiments described in the following.

## Results

To compare ACO-HPPFP-3 with algorithms for the 2D and 3D HP Protein Folding Problem described in the literature, we tested it on a number of standard benchmark instances as well as on two newly created data sets, one of which was obtained by randomly generating amino acid sequences with hydrophobicity value characteristic of globular proteins, while the other consists of biological sequences that were translated into HP strings using a standard hydrophobicity scale. (These new data sets will be described in more detail later in this section.)

### Results for standard benchmark instances

The 21 standard benchmark instances for 2D- and 3D-HP protein folding shown in Table 1 have been widely used in the literature [6,12,14-17,19,25]. Experiments on these standard benchmark instances were conducted by performing a number of independent runs for each problem instance (in 2D: 500 runs for sequence length *n *≤ 50, 100 runs for 50 <*n *≤ 64, and 20 runs for *n >*64; in 3D: 100 runs for each sequence). Unless explicitly indicated otherwise, we used the following parameter settings for all experiments: *α*: = 1, *β*: = 2, *ρ*: = 0.8 and *θ*: = 0.05. Furthermore, all pheromone values were initialised to 1/3 in 2D and to 1/5 in 3D, and a population of 100 ants was used, 50% of which were allowed to perform local search. The local search procedure was terminated when no improvement in energy had been obtained after between 1 000 (for *n *≤ 50) and 10 000 (for *n *> 50) scans through the protein sequence. We used an elitist pheromone updating scheme in which only the best 1% of all ants was allowed to perform pheromone updates. The probability 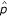 of keeping the previous direction when feasible during the long-range mutation move was set to 0.5 (see 'Methods' section). These settings were determined in a series of experiments in which we studied the influence of different parameter settings and will be further discussed later. All experiments were performed on PCs with 2.4 GHz Pentium IV CPUs, 256 Kb cache and 1 MB RAM, running Redhat Linux (our reference machine), and run-time was measured in terms of CPU time.

Most studies of EA and MC methods in the literature, including [12,14,15,19], report the number of valid conformations scanned during the search. This makes a performance comparison difficult, since run-time spent for backtracking and the checking of partial or infeasible conformations, which may vary substantially between different algorithms, is not accounted for. We therefore compared ACO to the best-performing algorithm from the literature for which performance data in terms of CPU time is available – PERM [18] (we used the most recent implementation, which was kindly provided by P. Grassberger). We note that the most efficient PERM variant for the HP Protein Folding Problem uses an additional penalty of 0.2 for H-P contacts [34]. Since this corresponds to an energy function different from that of the standard HP model underlying our ACO algorithm as well as other algorithms developed in literature, we used the best performing variant of PERM [18] based on the standard energy function in our experiments. It may be noted that the chain growth process in PERM can start from the *N*- or *C*-terminus of the given HP sequence, and in many cases, this results in substantial differences in the performance of the algorithm. To capture this effect, we always ran PERM in both directions, and in addition to the respective average run-times, *t*_1 _and *t*_2_, we report the expected time for solving a given problem instance when performing both runs concurrently, *t*_*exp *_= 2·(1/*t*_1 _+ 1/*t*_2_)^-1^. For all runs of PERM, the following parameter settings were used: inverse temperature *γ*: = 26 and *q*: = 0.2.

The results obtained on standard 2D benchmark instances (see Table 2) indicate that ACO-HPPFP-3 is competitive with the EA and MC methods described in the literature; it works very well on sequences of sizes up to 64 amino acids and produces high quality suboptimal configurations for the longest sequences considered here (85 and 100 amino acids). On average, ACO requires less CPU time than PERM for finding best known conformations for Sequence S1-8; but PERM performs better for Sequences S1-6 and S1-7 as well as for the longer sequences of 85 to 100 residues (Sequence S1-9 to S1-11).

**Figure 4:**
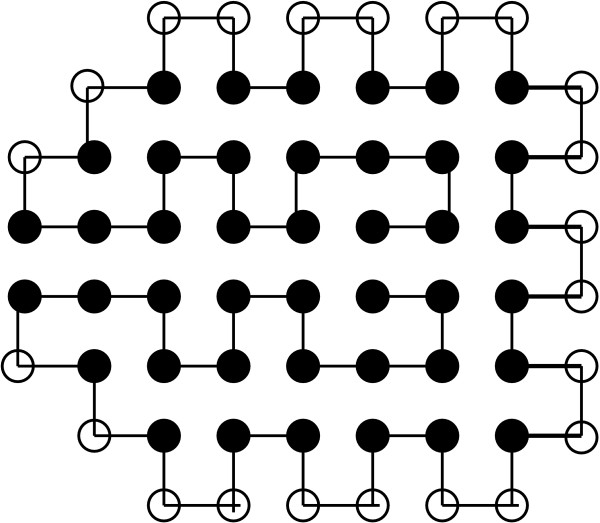
**The 2D native state of the standard Sequence S1-8**. The native conformation of Sequence S1-8 from Table 1 (64 amino acids; energy -42), found by ACO-HPPFP-3 in an average CPU time of 1.5 hours and by PERM in *t*_1 _= *t*_2 _= *t*_*exp *_= 78 hours.

Sequence S1-8 has a very symmetrical optimal state (see Figure 4), which – as argued in [18] – would be difficult to find for any chain growing algorithm. All algorithms from the literature which we are aware of have problems folding this sequence; ACO-HPPFP-3, on the other hand, is able to handle this instance quite well, since a number of ants folding from different starting points in conjunction with a local search procedure that involves large-scale mutations originating from different sequence positions can produce good partial folds for various parts of the chain. In comparison with other algorithms for the 2D HP Protein Folding Problem considered here (EA, EMC, MSOE), ACO-HPPFP-3 generally shows very good performance on standard benchmark instances.

In case of the 3D HP Protein Folding Problem (see Table 3), the majority of algorithms for which we were able to find performance results in the literature use heuristics that are highly specialised for this problem. Unlike HZ, CG and CI, ACO-HPPFP-3 finds optimal (or best known) solution qualities for all sequences. However, PERM (when folding from the *N*-terminus) and CHCC consistently outperform ACO-HPPFP-3 on these standard 3D HP benchmark instances, and CG reaches best known solution qualities substantially faster in many cases. We note that for Sequence S2-3 and S2-7, PERM'S performance is greatly dependent on the folding direction.

### Result for new biological and random data sets

To thoroughly test the performance of ACO-HPPFP-3, we created two new data sets of random and biological sequences of length ≈ 30 and ≈ 50 amino acids (ten sequences for each length; for details, see additional data file 1). Random sequences were generated based on the observation that most globular proteins have a fairly uniform amino acid profile, and the percent of hydrophobic residues of majority of globular proteins falls in the range of 40–50% [35]. Thus, the probability of generating character *H *at each position of a sequence was chosen to be 0.45, and in the remaining cases (*i.e., *with probability 0.55), we generated a P.

For the biological test-sets, ten sequences were taken from the PDBSELECT data set with homology < 25% from the Protein Data Bank (PDB) in order to obtain a non-redundant representative set of proteins. These protein sequences were translated into HP strings using the hydrophobicity scale classification of RASMOL [36], according to which the following amino acids were considered hydrophobic: *Ala*, *Leu*, *Val*, *Ile*, *Pro*, *Phe*, *Met*, *Trp*, *Gly *and *Tyr*. Non-standard amino acid symbols, such as X and Z, were skipped in this translation.

**Figure 5:**
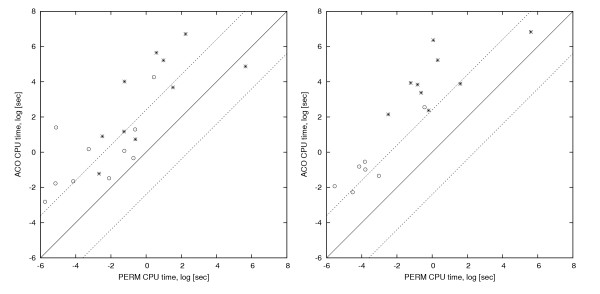
**Performance comparison of ACO-HPPFP-3 and PERM on biological and random instances in 2D**. Mean CPU time (natural log transformed) required by ACO-HPPFP-3 *vs *PERM for reaching the best solution quality, as observed over 10 runs with a cut-off time of 1 CPU hour for sequences of length 30 and 50 in 2D. The left and right plots show the results for the biological and random test-sets, respectively. Performance results for instances of size 30 are indicated by circles, while stars mark results for instances of size 50. The dashed lines indicate the band within which performance differences are not statistically significant. Mean run-times were obtained from 10 runs per instance and algorithm, and we only show data points for the runs where the best known solution quality was reached at least in some runs out of 10 by both algorithms (when unsuccessful runs were present, the expected time was calculated as in [43]); results for both successful and unsuccessful runs are given in the Additional file 1.

**Figure 6:**
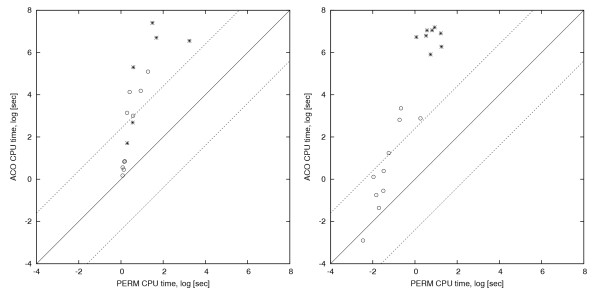
**Performance comparison of ACO-HPPFP-3 and PERM on biological and random instances in 3D**. Mean CPU time (natural log transformed) required by ACO-HPPFP-3 *vs *PERM for reaching the best solution quality, as observed over 10 runs with a cut-off time of 1 CPU hour for sequences of length 30 and 50 in 3D. The left and right plots show the results for the biological and random test-sets, respectively. Performance results for instances of size 30 are indicated by circles, while stars mark results for instances of size 50. Mean run-times were obtained from 10 runs per instance and algorithm, and we only show data points for the runs where the best known solution quality was reached at least in some runs out of 10 by both algorithms (when unsuccessful runs were present, the expected time was calculated as in [43]); results for both successful and unsuccessful runs are given in the Additional file 1.

Figures 5 and 6 illustrate the performance of ACO-HPPFP-3 *vs *PERM in terms of mean CPU time over 10 runs per instance and algorithm; for practical reasons, each run was restricted to 1 CPU hour on our reference machine, and the lowest energies obtained in these runs (listed in additional data file 1) are not necessarily optimal.

As can be seen from these results, in 2D, ACO-HPPFP-3 performs roughly comparably to PERM (PERM'S *t*_*exp *_was calculated as described in the previous subsection): ACO-HPPFP-3 reaches the same energies as PERM, but on some instances, particularly of length 50, requires more run-time. In 3D, ACO-HPPFP-3 generally requires a comparable amount of run-time on sequences of length 30 and outperforms PERM on one random sequences of length 30, but performs noticeably worse on sequences of length 50 and in some cases does not reach the same energy. We also generated longer sequences of length 75; for these, ACO-HPPFP-3 failed to reach the minimal energy values obtained by PERM in a number of cases. The run-times for both algorithms are reported in detail in Additional file 1; we note that on some sequences, the performance of PERM depends significantly on the direction of folding. Interestingly, there is no significant difference in performance between the biological and random test-sets for either PERM or ACO-HPPFP-3.

In summary, the performance of ACO-HPPFP-3 is comparable with that of PERM (the best known algorithm for the 2D and 3D HP Protein Folding Problem) on biological and random sequences of length 30–50, but worse on longer sequences. This scaling effect is significantly more pronounced in 3D than in 2D. We note that neither ACO-HPPFP-3 nor PERM were optimised for short sequences (*n *≤ 30), but by using parameter settings different from the ones specified earlier, the performance of both algorithms can be significantly improved in this case.

### Characteristic performance differences between ACO and PERM

**Figure 7:**
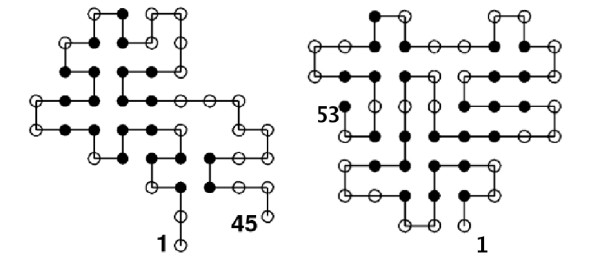
**Illustration and comparison of difficult structures for PERM and ACO-HPPFP-3 in 2D**. Left side: Lowest energy conformation of a biological sequence (B50-7, 45 amino acids, energy -17) that is harder for PERM (*t*_1 _= 271, *t*_2 _= 299, *t*_*exp *_= 284 CPU seconds) than for ACO-HPPFP-3 (*t*_*exp *_= 130 CPU seconds; cut-off time 1 CPU hour). Right side: Lowest energy conformation of a biological sequence (B50-5, 53 amino acids, energy -22) that is much harder for ACO-HPPFP-3 than for PERM; within a cut-off time of 1 CPU hour, both ACO-HPPFP-3 and PERM reached this energy in 10 out of 10 runs in *t*_*avg *_= 820 and *t*_1 _= 5, *t*_2 _= 118, *t*_*exp *_= 9 CPU seconds on average, respectively.

To further investigate the conditions under which ACO performs well compared to PERM, we visually examined native conformations found by both algorithms, paying special attention to conformations for which one of the two algorithms does not perform well (see Figures 7 and 9). Based on our observations, we hypothesised that PERM usually performs well on sequences that have a structural nucleus in the native conformation at one of the ends of the sequence (particularly the end from which PERM starts folding the sequence); on the other hand, it has trouble folding sequences whose native conformations have structural nuclei in the middle of the sequence. In comparison, ACO is not significantly affected by the location of the structural nucleus (or multiple nuclei) in the sequence, since it uses construction from different folding points as well as the long-range mutation moves in local search, which can initiate refolding from arbitrary sequence positions. Here, we use the term 'structural nucleus' to refer to a predominantly locally folded part of the chain that can be relatively easily folded sequentially based on local sequence information [37]. For most sequences considered in this study, we observed a single structural nucleus, which is not surprising, given their relatively short length; however, it is generally believed that longer sequences have multiple folding nuclei [37].

The left side of Figure 7 shows an example of a relatively short biological sequence (B50-7, 45 amino acids) with a unique native hydrophobic core in the 2D HP model. (This is rare for HP sequences, which usually have a high ground state and hydrophobic core degeneracy: According to our observations, of the 11 standard benchmark instances in 2D, only Sequences S1-1, S1-3, S1-4 have a unique hydrophobic core; in 3D, none of the sequences studied here have a unique hydrophobic core.) This sequence has no structural nuclei at its ends; instead, the two ends interact with each other. ACO-HPPFP-3 outperforms PERM by a factor of 2 on this sequence in terms of CPU time: using a cut-off time of 1 CPU hour per run, PERM found the optimum with energy -17 in an average run-time of 284.06 CPU seconds (*t*_1 _= 271 sec, *t*_2 _= 299 sec), while using the same cut-off time and machine, ACO-HPPFP-3 found the optimum in an average run-time of 130 CPU seconds.

**Figure 8:**
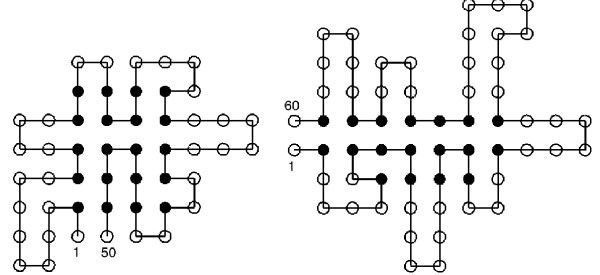
**Performance of ACO-HPPFP-3 and PERM on designed sequences in 2D HP**. Left side: Unique minimal energy conformation of a designed sequence, D-1 (length 50, energy -19); ACO-HPPFP-3 reaches this conformation much faster than PERM when folding from the left end (mean run-time over 100 successful runs for ACO-HPPFP-3: 236 CPU seconds, compared to *t*_1 _= 3 795, *t*_2 _= 1, *t*_*exp *_= 2 CPU seconds for PERM). Right side: Unique native conformation of another designed sequence, D-2 (length 60, energy -17); ACO-HPPFP-3 finds this conformation much faster than PERM folding from either end (mean run-time over 100 successful runs for ACO-HPPFP-3: 951 CPU seconds, compared to *t*_1 _= 9 257, *t*_2 _= 19 356, *t*_*exp *_= 12 524 CPU seconds for PERM).

We also designed two additional sequences, D-1 and D-2, of length 50 and 60, respectively, that have a unique native state in which both ends of the sequence interact with each other (see Figure 8). Sequence D-1 also has a structural nucleus near its *C*-terminus. When testing the performance of PERM and ACO-HPPFP-3 on these sequences, we found that on D-1, ACO-HPPFP-3 requires a mean run-time of 236 CPU seconds, compared to *t*_1 _= 3 795, *t*_2 _= 1, *t*_*exp *_= 2 CPU seconds for PERM (values are based on 100 successful runs). When this sequence was reversed, PERM started folding the sequence from the structural nucleus, and its mean run-time dropped to 1 CPU second. A result similar to that for sequence B50-7 was obtained for Sequence D-2, which has no structural nuclei at the ends, but a native state in which the ends interact with each other. Here, ACO-HPPFP-3 was found to require a mean run-time of 951 CPU seconds (again, mean run-times were obtained from 100 successful runs), compared to *t*_1 _= 9 257, *t*_2 _= 19 356, *t*_*exp *_= 12 525 CPU seconds for PERM; as expected, in this case, reversing the folding order of the sequence did not cause a decrease in PERM'S run-time.

We also analysed native conformations of sequences on which PERM outperforms ACO and observed that the end from which PERM starts folding is relatively compact and forms a structural nucleus in the resulting conformation.

**Figure 9:**
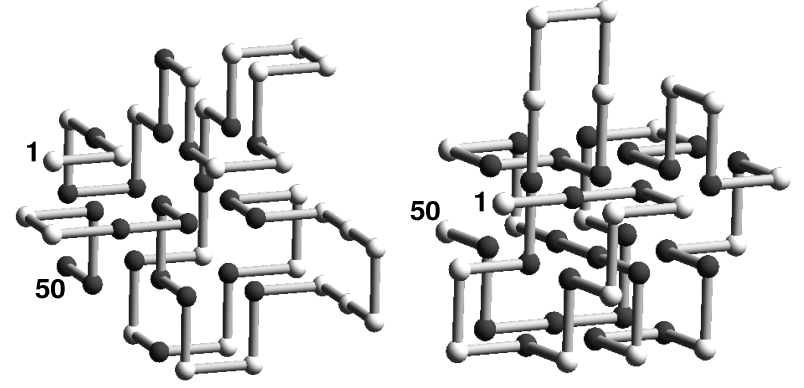
**Illustration and comparison of difficult structures for PERM and ACO-HPPFP-3 in 3D**. Left side: Lowest energy conformation of random sequence R50-9 (50 amino acids, energy -30), which is harder for PERM when folding from the left end than for ACO-HPPFP-3; with a cut-off time of 1 CPU hour, ACO-HPPFP-3 reached this energy in 10 out of 10 runs with *t*_*exp *_= 1000 CPU seconds, while PERM failed to find a conformation with this energy in 7 out of 10 runs when folding from the left end (*t*_1 _= 9 892, *t*_2 _= 2, *t*_*exp *_= 3 CPU seconds). Right side: Lowest energy conformation of random sequence R50-7 (50 amino acids, energy -38), which is much harder for ACO-HPPFP-3 than for PERM; with a cut-off time of 1 CPU hour, PERM reached this energy in two out of 10 runs when folding from the left and in 10 of 10 runs when folding from the right end in *t*_1 _= 15 322, *t*_2 _= 46, *t*_*exp *_= 92 CPU seconds, while the lowest energy reached by ACO-HPPFP-3 over ten runs was -37.

An example of a conformation with the structural nucleus at the beginning of the sequence (near the *N*-terminus, *i.e., *residue 1) is shown in the right panel of Figure 7. For this biological sequence (B50-5, 53 amino acids), PERM finds an optimal conformation with an energy of -22 in *t*_1 _= 5, *t*_2 _= 118, *t*_*exp *_= 9 CPU seconds, while the average run-time for ACO-HPPFP-3 is 820 CPU seconds. Our ACO algorithm generally performs worse than PERM on sequences that have structural nuclei at the ends, because it tends to spend substantial amounts of time compacting local regions in the interior of the sequence, while PERM folds more systematically from one end. These observations also hold in 3D, as seen from two random sequences folded in 3D (see Figure 9).

To further investigate our hypothesis, we studied differences between the distributions of native conformations found by ACO-HPPFP-3 and PERM, respectively. For this purpose, we introduced the notion of *relative H-H contact *order, which captures arrangement of H residues in the core of the folded protein, and thus determines the topology of the conformation (the closely related concept of contact order was first defined in [38]). Relative H-H contact order is defined as follows:


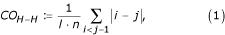


where *l *is the number of H-H contacts, *n *is the number of H residues in the sequence, and *i *and *j *are interacting H residues that are not neighbours in the chain. Intuitively, *CO*_*H-H *_specifies the average sequence separation between H-H residues in contact per H in the sequence.

**Figure 10:**
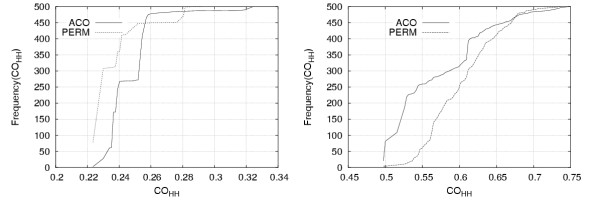
**Comparison of distributions of H-H contact order of native structures found by ACO-HPPFP-3 and PERM in 2D and 3D**. Distributions of H-H contact order for 500 conformations of Sequence S1-7 from Table 1 (60 amino acids) in 2D (left side) and Sequence S1-5 from Table 1 (48 amino acids) in 3D (right side) found by ACO-HPPFP-3 and PERM.

Figure 10 shows cumulative frequency distributions of relative H-H contact order values for sets of native conformations of a 2D (the left panel) and 3D (the right panel) standard benchmark instance, respectively, found by ACO-HPPFP-3 and PERM over 500 independent runs, each of which was terminated as soon as a native conformation had been found. These results show that the ACO algorithm finds a set of native conformations with a wider range of H-H contact order values than PERM; in particular, ACO-HPPFP-3 finds conformations with high relative H-H contact oder as compared to PERM (more distant parts of the chain interact; for example, relative *CO*_*H-H *_= 0.324 for Sequence S1-7 in 2D and relative *CO*_*H-H *_= 0.75 for Sequence S2-5 in 3D are not found by PERM; similar results were obtained for other sequences), which further supports our hypothesis that both, in 2D and 3D, PERM is biased toward a more restricted set of native conformations. We performed analogous experiments for the case where PERM is allowed to keep certain statistics from one run to another as in [18] (runs are no longer independent) and found no significant differences in the set of conformations obtained (data not shown).

**Figure 11:**
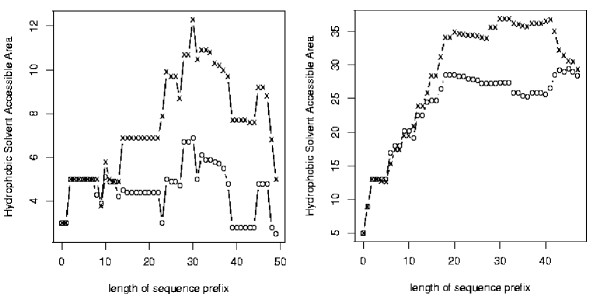
**Plot of mean hydrophobic solvent accessible area, ACO-HPPFP-3 *vs *PERM in 2D and 3D**. Mean hydrophobic solvent accessible area as a function of prefix length for a biological sequence (B50-4, 50 amino acids) in 2D (left side) and Sequence S2-6 from Table 1 (48 amino acids) in 3D. Crosses and circles represent mean values for an ensemble of 100 native structures found by ACO-HPPFP-3 and PERM, respectively.

**Figure 12:**
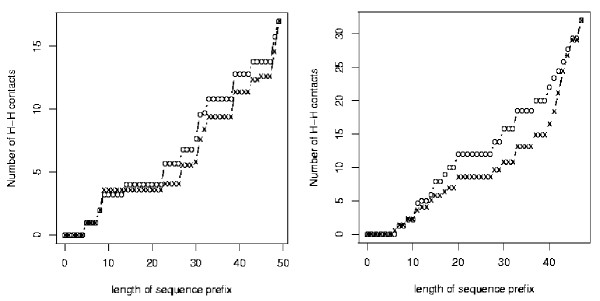
**Plot of mean number of H-H contacts, ACO-HPPFP-3 *vs *PERM in 2D and 3D**. Mean number of H-H contacts as a function of prefix length for a biological sequence (B50-4, 50 amino acids) in 2D (left side) and Sequence S2-6 from Table 1 (48 amino acids) in 3D. Crosses and circles represent mean values for an ensemble of 100 native structures found by ACO-HPPFP-3 and PERM, respectively.

**Figure 13:**
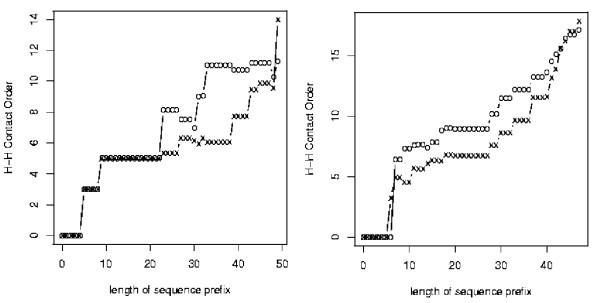
**Plot of mean H-H contact order, ACO-HPPFP-3 *vs *PERM in 2D and 3D**. Mean H-H contact order as a function of prefix length for a biological sequence (B50-4, 50 amino acids) in 2D (left side) and Sequence S2-6 from Table 1 (48 amino acids) in 3D. Crosses and circles represent mean values for an ensemble of 100 native structures found by ACO-HPPFP-3 and PERM, respectively.

To further examine the topological differences between ensembles of native conformations found by the two algorithms, we also looked at the hydrophobic solvent accessible area (defined as *SA*_*H-H*_: = ∑_*h*_*E*_*h*_, where *E*_*h *_is the number of unoccupied lattice sites around H residue *h*), the number of H-H contacts, and the H-H contact order as a function of the length of the sequence prefix (starting from the *N*-terminus of the sequence – where PERM starts folding). In this analysis, we calculated the properties of interest mentioned above for the native conformations found in 100 independent runs by ACO-HPPFP-3 and PERM, and plotted the mean values of the respective quantities as functions of the sequence prefix length (see Figures 11, 12 and 13).

As seen in Figure 11, ACO-HPPFP-3 is less greedy than PERM, both in 2D (left side) and in 3D (right side), and it tends to leave more lattice sites around H residues accessible for future contacts with other H residues that appear later in the chain. This is also reflected in the mean number of H-H contacts formed when folding prefixes of increasing length; ACO-HPPFP-3 tends to form fewer H-H contacts than PERM for short and medium size prefixes (see Figure 12). By examining the dependence of absolute H-H contact order (defined as 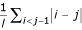, the average sequence separation per H-contact) on prefix length, we furthermore observed that different from PERM, ACO-HPPFP-3 realises the bulk of its local H-H interactions in the middle of the given sequence (see Figure 13). This further confirms that ACO is capable of finding native conformations with structural folding nuclei that are not located at or near the end of a given protein sequence. The results illustrated in Figures 11, 12 and 13 are typical for all 2D and 3D HP instances we studied.

## Discussion

Although conceptually rather simple, our ACO algorithm is based on a number of distinct components and mechanisms. A natural question to ask is whether and to which extent each of these contributes to the performance reported in the previous section. A closely related questions concerns the impact of parameter settings on the performance of ACO-HPPFP-3; further details concerning parameters can be found in the 'Methods' section. To address these questions, we conducted several series of experiments. In this context, we primarily used three standard test sequences: Sequence S1-7 of length 60 and Sequence S1-8 of length 64 (long sequences) in 2D, as well as Sequence S2-5 of length 48 in 3D (all standard benchmark sequences for 3D are 48 amino acids in length); these sequences were chosen because the CPU time required to find the best known solutions was sufficiently small to perform a large number of runs (100–200 per instance).

Following the methodology of Hoos and Stützle [39], we measured run-time distributions (RTDs) of our ACO algorithm, which represent the (empirical) probability distribution over the run-time required to reach (or exceed) a given solution quality; the solution qualities used here are the known optima or best known energies for the respective sequences.

### Pheromone values and heuristic information

Two important components of any ACO algorithm are the heuristic function, which indicates the desirability of using particular solution components during the construction phase, and the pheromone values, which represent information learned over multiple iterations of the algorithm. Three parameters control the influence of the pheromone information versus heuristic information on the construction of candidate solutions: the relative weight of the pheromone information, *α*; the relative weight of the heuristic information, *β*; and the pheromone persistence, *ρ *(see also 'Methods' section).

**Figure 14:**
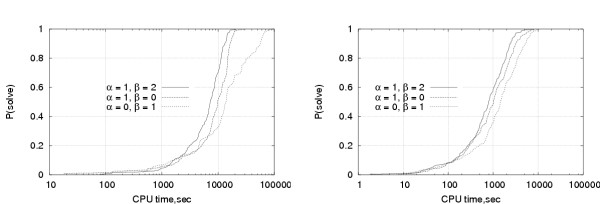
**Impact of parameter settings on ACO-HPPFP-3 performance in 2D and 3D: relative weights of pheromone and heuristic information**. Effect of the relative weights of pheromone information, *α*, and heuristic information, *β*, on the average CPU time required for obtaining minimal energy conformations of Sequence S1-8 in 2D (length 64, left side) and Sequence S2-5 in 3D (length 48, right side).

In the first experiment, we investigated the impact of pheromone (*α*) and heuristic information (*β*), and their relative importance for the performance of our ACO algorithm. As can be seen from the results shown in Figure 14, both the pheromone values and the heuristic information are important in 2D and 3D; when ignoring either of them (*α*: = 0 or *β*: = 0, respectively), the algorithm performs worse, particularly for longer 2D sequences (*n *> 50; for short 2D sequences with *n *≤ 50, the pheromone matrix does not appear to play a significant role, since sequences are generally easily solved by the subsidiary local search procedure alone). The optimal settings for *α *and *β *for most problem instances seem to be around *α *= 1 and *β *= 2, as shown in Figure 14. It should be noted that in 3D, pheromone information appears to be less important than in 2D, which suggests that the specific solution components used in our algorithms are somewhat less meaningful in 3D.

**Figure 15:**
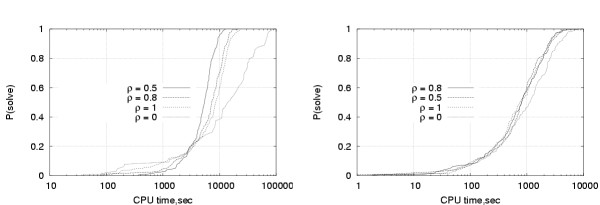
**Impact of parameter settings on ACO-HPPFP-3 performance in 2D and 3D: pheromone persistence**. Effect of the pheromone persistence parameter, *ρ*, on the average CPU time required for obtaining minimal energy conformations of Sequence S1-8 in 2D (length 64, left side) and Sequence S2-5 in 3D (length 48, right side).

The goal of the next experiment was to further explore the role of experience accumulated over previous iterations in the form of pheromone values. To this end, we varied the pheromone persistence, *ρ*, while keeping other parameters constant. The results shown in Figure 15 show that in 2D, it is important to utilise past experience (*i.e., *to choose *ρ *> 0), but also to weaken its impact over time (*i.e., *to use *ρ *< 1). At the same time, closer examination revealed that for *ρ *> 0, attrition, *i.e., *the construction of inextensible partial conformations, is a major problem, which is a result of the accumulation of pheromone from multiple conformations. This is why the backtracking mechanism described in the 'Methods' section is extremely important for the performance of our algorithm in 2D. In 3D, for the previously stated reasons and because of the fact that the attrition problem is much less severe, the impact of the persistence parameter is generally smaller than in 2D.

### Ant colony size and length of local search phase

**Figure 16:**
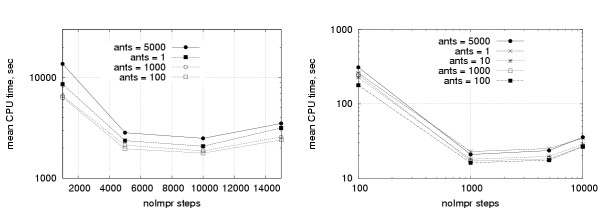
**Parameter settings influence on ACO-HPPFP-3 performance in 2D and 3D: ant colony size and maximum number of non-improving local search steps**. Mean CPU time required for finding minimum energy conformations of Sequence S1-7 in 2D (length 60, left side) and Sequence S2-5 in 3D (length 48, right side), as a function of ant colony size and the maximum number of non-improving local search steps.

During the initial empirical evaluation of our algorithm, we observed that ant colony size (*i.e., *the number of ants used in each iteration) and the duration of local search (expressed as a number of non-improving search steps we are willing to consider before terminating the local search procedure) are correlated and significantly affect its performance. To further investigate this phenomenon, we conducted additional experiments in which we fixed the ant colony size and varied the maximal number of non-improving steps during local search, and vice versa. In this series of experiments, different colony sizes were considered, from a single ant up to a population of 5 000 ants, and the number of non-improving steps in local search was varied from 100 to 10 000. The results, shown in Figure 16, indicate that there is an optimal colony size of about 100 ants for both, 2D and 3D; ACO-HPPFP-3 is quite robust with respect to colony size, but performance decreases for very small or very large colony sizes. Intuitively, this is the case because the use of a population of ants provides diversification to the search process, which enables it to explore different regions of the underlying search space; very small populations provide insufficient diversification, and the search stagnates easily, while for very large populations, the additional time required for running the search phases for each ant on the same sequential machine is not amortised any longer by increased efficiency of the overall search process.

Our results also indicate that the performance of ACO-HPPFP-3 is more sensitive to the number of non-improving steps than to ant colony size. The optimal value for the maximum number of non-improving steps tolerated (per ant) before the local search phase terminates was found to be around 1 000 for short 2D sequences (*n *≤ 50) and around 10 000 for long 2D sequences (*n *> 50); the latter value also appeared to be optimal for all 3D sequences considered here. This observation follows the intuition that more degrees of freedom, as present for longer sequences and in higher dimensions, require more time for local optimisation, since for any conformation, improving neighbours tend to be rarer and hence harder to find.

### Selectivity and persistence of local search

As described in the 'Methods' section, our ACO algorithm uses selective local search, *i.e., *local search is only performed on a certain fraction of the lowest energy conformations. We observed that ACO-HPPFP-3 is fairly robust with respect to the fraction of conformations to which local search is applied; good performance was obtained for local search selectivity values between 5% and 50%, but performance was found to deteriorate when local search is performed by all ants. Intuitively, similar to colony size, local search selectivity has an impact on search diversification. If too few ants perform local search, insufficient diversification is achieved, which typically leads to premature stagnation of the search process. On the other hand, if local search is performed by too many ants, the resulting substantial overhead in run-time can no longer be amortised by increased search efficiency.

Similarly to selective local search, pheromone update was performed only by a certain fraction of so-called 'elitist ants' whose solution quality after the local search phase is highest within the population. As in the case of local search selectivity, ACO-HPPFP-3 shows robustly high performance for elitist fractions between 1% and 50% (results are not shown here), but performance deteriorates markedly when all ants in the colony are allowed to update the pheromone matrix.

**Figure 17:**
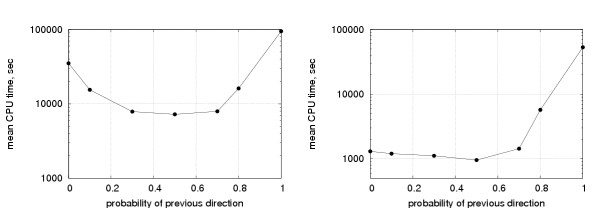
**Parameter settings influence on ACO-HPPFP-3 performance in 2D and 3D: probability of retaining previous directions in local search**. Mean CPU time required for finding minimum energy conformations of Sequence S1-8 in 2D (length 64, left side) and Sequence S2-5 in 3D (length 48, right side), as a function of the probability of retaining previous directions (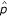) during long-range mutation moves.

In a final experiment, we studied the impact of the persistence of local search, *i.e., *of the probability 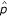 of retaining (feasible) previous relative directions during long-range mutation moves. As can be seen in Figure 17, good performance is generally obtained for 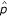 values between 0.3 and 0.7. Both extreme cases, 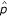 = 0, which corresponds to an extremely H-contact greedy mutation operator, and 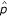 = 1, in which refolding always follows previous directions when feasible, result in a substantial decrease in performance. When 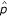 = 0, the decrease of performance in 3D is smaller than in 2D, because there is no severe attrition as in 2D, where greedy placement of H residues leads to early formation of very compact partial conformations, which often cannot be extended into valid complete conformations. The performance decrease for high 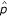 values is due to insufficient ability of the chain to fold into a new conformation that accommodates well the local change in structure which triggered the refolding.

## Conclusions

In this work, we have shown that ant colony optimisation (ACO) can be applied in a rather straight-forward way to the 2D and 3D HP Protein Folding Problems. Even though our ACO-HPPFP-3 algorithm is based on very simple structure components (single relative directions) and a simple subsidiary local search procedure (iterative first improvement), it performs fairly well compared to other algorithms and specialised heuristics on the benchmark instances considered here, particularly in 2D. The only non-specialised algorithm that typically performs better than our ACO algorithm, both in 2D and 3D, is PERM. We observed that, particularly in 3D, the run-time required by ACO-HPPFP-3 for finding minimum (or best known) energy conformations scales worse with sequence length than PERM. However, our results show that our ACO algorithm finds a different ensemble of native conformations compared to PERM, and has less difficulty folding sequences whose native states contain structural nuclei located in the middle rather than at the ends of a given sequence, as well as sequences with structures in which the ends interact. We found that two major components of ACO-HPPFP-3 – the pheromone values, which capture experience accumulated over multiple iterations of the search process and from multiple conformations, as well as the heuristic information that provides myopic guidance to the folding process – play a significant role for longer 2D sequences and, to a lesser extent, for 3D sequences, which suggests that in 3D, it may be preferable to associate pheromone values with more complex solution components.

We also found that the subsidiary local search procedure is crucial for the performance of the algorithm; in particular, to ensure that high-quality conformations are obtained, it is very important to allow the local search procedure to run sufficiently long. In an earlier version of our algorithm [7], we used substantially more stringent termination criteria, which forced us to additionally use non-greedy local search (probabilistic iterative improvement, which accepts worsening steps) in addition to the greedy local search procedure used here. The results presented in this study indicate that by using a new and simpler local search procedure, ACO-HPPFP-3 achieves better performance; this is probably due to the fact that the new local search procedure is based on a type of long-range move that leads to a larger effective search neighbourhood.

We have shown that all components of our ACO algorithms contribute to its performance. In particular, its performance is affected by the following components and parameters (listed in the order of decreasing impact): pheromone values, termination criterion for local search, persistence of long-range moves, ant colony size, pheromone persistence, heuristic function, selectivity of local search, and selectivity of pheromone update (*i.e., *fraction of elitist ants).

Because of its ability to find more balanced ensembles of minimum (or close to minimum) energy conformations, our new ACO algorithm can greatly facilitate investigations of the topology and location of structural nuclei, which we plan to undertake in future work. Finally, while HP protein folding problems are of considerable interest because of their conceptual simplicity, ultimately, most applications of protein folding algorithms require the use of more realistic models of protein structure. Since it does not rely on heuristics and properties that are specific to the HP model and yet performs very well on this restrictive, but not entirely unrealistic abstract model, we believe that relatively straight-forward extensions of our ACO algorithm to more complex and realistic models of protein structure hold significant promise.

## Methods

Our new ACO algorithm, ACO-HPPFP-3, iterates construction, local search, and pheromone update phases until a termination condition is satisfied; in the context of this work, we mostly terminated the algorithm upon reaching a given energy threshold. In the following, we describe the three search phases in detail.

### Construction phase, pheromone and heuristic values

During the construction phase of ACO-HPPFP-3, each ant first determines a starting point within the given protein sequence; this is done by uniform random choice. From this starting point, the sequence is folded in both directions, adding one residue at a time. Each ant performs probabilistic chain-growth construction of the protein conformation, where in every step, the structure is extended either to the left or to the right, such that the ratio of unfolded residues at each end of the protein remains (roughly) unchanged.

Here, we assume that folding is performed in 3D (the 2D case is handled analogously by considering three relative directions {*S, L, R*} instead of five {*S, L, R, U, D*}, see also [6]). The relative directions in which the conformation is extended in each construction step are determined probabilistically based on a heuristic function *η*_*i*,*d *_and pheromone values *τ*_*i*,*d*_, according to the formula:


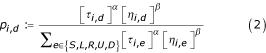


The pheromone values *τ*_*i*,*d *_indicate the desirability of using the local structure motif with relative direction *d *∈ {*S, L, R, U, D*} at sequence position *i*. Initially, all *τ*_*i*,*d *_are equal, such that local structure motifs are chosen in an unbiased way; but throughout the search process, the pheromone values are updated to bias folding towards the use of local motifs that occur in low-energy structures (the updating mechanism will be described in more detail later). The heuristic values *η*_*i*,*d *_are based on the energy function *E*. They are defined according to the Boltzman distribution as *η*_*i*,*d*_: = 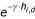, where *γ *is a parameter called the inverse temperature (as in [18]), and *h*_*i*,*d *_is the number of new H-H contacts achieved by placing amino acid *i *at the position specified by direction *d*.

During construction, it may happen that the chain cannot be extended without running into itself. This situation is called *attrition*, and our algorithm overcomes it as follows: First, starting at the end at which attrition occurred, half of the sequence that has been folded up to this point is unfolded. Then, this segment of the chain is refolded; the first residue (*i.e., *the last one that was unfolded) is placed such that its relative direction differs from what it had been when attrition occurred, while all of the subsequent residues are folded in a feasible direction that is chosen uniformly at random. This backtracking mechanism is particularly important for longer protein sequences in 2D, where infeasible conformations are frequently encountered during the construction phase.

### Local search

The local search phase is based on a long-range mutation move that has been designed to avoid infeasible conformations. It also has a number of important advantages over the more commonly used point mutation moves or Monte Carlo moves (*i.e., *the end, crankshaft and corner moves [40]): It is easy to implement; it decreases the number of infeasible conformations encountered, even when the protein is very compact (at high densities); it considers a larger neighbourhood that subsumes the single point mutation neighbourhood; and it has some validity in terms of the physical processes taking place during the protein folding process. Similar attempts have been previously undertaken, but these involved disconnection of the chain [21].

**Figure 18:**
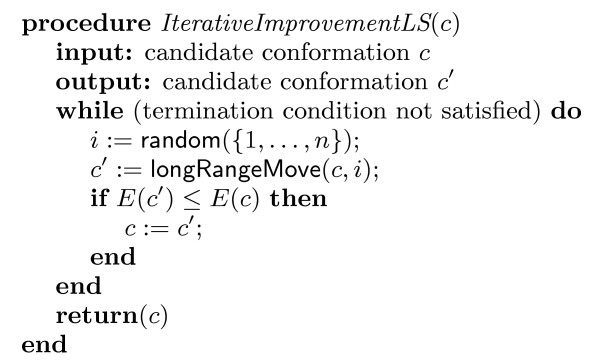
**Outline of the subsidiary local search procedure**. The iterative first improvement local search procedure that is performed by selected ants after the construction phase.

From studies of protein folding dynamics, it is known that proteins display a broad range of motions that range from localised motions to slow large-scale movements [37]. Inspired by this complex process, we designed a long-range mutation move that starts by selecting a residue whose relative direction is randomly mutated and then adapts the rest of the chain by probabilistically changing relative directions starting from this initial position [7]. During this adaptation, for each residue, with a probability 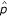 (0 ≤ 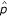 ≤ 1) its previous relative direction, if it is still feasible, is left unchanged, and otherwise (*i.e., *with probability 1 - 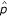, or if the previous direction has become infeasible), a different relative direction is chosen, where the probability for each direction *d *is proportional to the corresponding heuristic value *η*_*i*,*d*_. Formally, this can be written as follows:


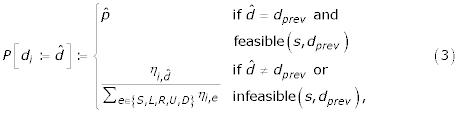


where *P*[*d*_*i *_: = 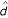] is the probability of choosing direction 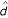 as the relative direction *d*_*i *_at sequence position *i*. Unlike in our previous implementation [7], the local search phase of our new ACO algorithm is a simple iterative first improvement procedure that is based on this long-range mutation move. The outline of this local search procedure is shown in Figure 18. Iterative first improvement accepts a new conformation generated via long-range mutation only if the solution quality of a new conformation *c*' improves over the current solution quality (energy) of *c. *This search process is greedy in the sense that it does not allow worsening steps, and it is terminated when no improving steps have been found after a specific number of scans through the chain (this number is a parameter of the algorithm). Since this local search procedure has a relatively high time-complexity, in each iteration of ACO-HPPFP-3 it is only applied to a certain fraction of the highest-quality conformations constructed by the ants in the preceding construction phase.

### Update of the pheromone values

After each construction and local search phase pheromones are updated according to

*τ*_*i*,*d*_:= *ρ*·*τ*_*i*,*d*_,     (4)

where 0 <*ρ *≤ 1 is the pheromone persistence, a parameter that determines how much of the information gathered in previous iterations is retained. Subsequently, selected ants with low-energy conformations update the pheromone values according to

*τ*_*i*,*d*_:= *τ*_*i*,*d *_+ Δ_*i*,*d*,*c*_,     (5)

where Δ_*i*,*d*,*c *_is the relative solution quality of the given ant's candidate conformation *c *if that conformation contains local structure motif *d *at sequence position *i*, and zero otherwise.

As a further mechanism for preventing search stagnation, we use an additional renormalisation of the pheromone values that is conceptually similar to the method used in *MAX - MIN* Ant System [41]: After the standard pheromone updates according to Equations 3 and 4, all *τ *values are normalised such that ∑_*d*∈{*S*,*L*,*R*,*U*,*D*} _*τ*_*i*,*d *_= 1 for every residue *i*; additionally, whenever for a given sequence position *i *the minimal normalised pheromone value (min_*d*∈{*S*,*L*,*R*,*U*,*D*} _*τ*_*i*,*d*_)/(∑_*d*∈{*S*,*L*,*R*,*U*,*Dr*} _*τ*_*i*,*d*_) falls below a threshold *θ *(which is a parameter of the algorithm), the minimal *τ*_*i*,*d *_value is set to *θ*, while the maximal *τ*_*i*,*d *_value is decreased by *θ *- min_*d*∈{*S*,*L*,*R*,*U*,*D*} _*τ*_*i*,*d*_. (If there is more than one minimal *τ*_*i*,*d *_value, all of these are increased to *θ*, and if there is more than one maximal *τ*_*i*,*d *_value, one of them is chosen uniformly at random.) This guarantees that the probability of selecting an arbitrary local structure motif for the corresponding sequence position does not become arbitrarily small, and hence ensures the probabilistic approximate completeness of our algorithm (see [42]).

### Implementation details and availability

ACO-HPPFP-3 has been implemented in C++ and compiled using gcc (version 3.3.3) for the Linux operating system; a Linux executable is available from .

## Authors' contributions

Both authors contributed to the development of ideas, design of experiments, analysis and interpretation of results, and the writing of the paper. AS implemented the proposed method and performed the computational experiments.

## Supplementary Material

Additional File 1 **Additional information on biological and randomly generated HP sequences. **This file (in .pdf format) contains tables providing additional information on our new test sets of biological and randomly generated HP sequences and the results from our computational experiment with ACO and PERM. Click here for file

## Figures and Tables

**Table 1:** 2D and 3D HP standard benchmark instances. Benchmark instances for the 2D and 3D HP Protein Folding Problem used in this study with optimal or best known energy values E*. Most instances for 2D and 3D HP can also be found in [44]; Sequence S1-9 (2D) is taken from [45], and the last two instances (2D) are from [21]. *H*_*i *_and *P*_*i *_indicate a string of *i *consecutive H's and P's, respectively; likewise, (*s*)_*i *_indicates an *i*-fold repetition of string *s*.

ID	Length	E*	Protein Sequence
*2D HP*			

S1-1	20	*-9*	*(HP)*_2_*PH*_2_*PHP*_2_*HPH*_2_*P*_2_*HPH*
S1-2	24	*-9*	*H*_2_(*P*_2_*H*)_7_*H*
S1-3	25	-8	*P*_2_*HP*_2_(*H*_2_*P*_4_)_3_*H*_2_
S1-4	36	-14	*P*_3_*H*_2_*P*_2_*H*_2_*P*_5_*H*_7_*P*_2_*H*_2_*P*_4_*H*_2_*P*_2_*HP*_2_
S1-5	48	-23	*P*_2_*H*(*P*_2_*H*_2_)_2_*P*_5_*H*_10_*P*_6_(*H*_2_*P*_2_)_2_*HP*_2_*H*_5_
S1-6	50	-21	*H*_2_(*PH*)_3_*PH*_4_*PH*(*P*_3_*H*)_2_*P*_4_*H*(*P*_3_*H*)_2_*PHPH*_4_(*HP*)_3_*H*_2_
S1-7	60	-36	*P*_2_*H*_3_*PH*_8_*P*_3_*H*_10_*PHP*_3_*H*_12_*P*_4_*H*_6_*PH*_2_*PHP*
S1-8	64	-42	*H*_12_(*PH*)_2_(*P*_2_*H*_2_)_2_*P*_2_*HP*_2_*H*_2_*PPH*_2_*P*_2_*HP*_2_(*H*_2_*P*_2_)_2_(*HP*)_2_*H*_12_
S1-9	85	-53	*H*_4_*P*_4_*H*_12_*P*_6_(*H*_12_*P*_3_)_3_*HP*_2_(*H*_2_*P*_2_)_2_*HPH*
S1-10	100	-50	*P*_3_*H*_2_*P*_2_*H*_4_*P*_2_*H*_3_(*PH*_2_)_2_*PH*_4_*P*_8_*H*_6_*P*_2_*H*_6_*P*_9_*HPH*_2_*PH*_11_*P*_2_*H*_3_*PH*_2_*PHP*_2_*HPH*_3_*P*_6_*H*_3_
S1-11	100	-48	*P*_6_*HPH*_2_*P*_5_*H*_3_*PH*_5_*PH*_2_*P*_4_*H*_2_*P*_2_*H*_2_*PH*_5_*PH*_10_*PH*_2_*PH*_7_*p*_11_*H*_7_*P*_2_*HPH*_3_*P*_6_*HPH*_2_

*3D HP*			

S2-1	48	-32	*HPH*_2_*P*_2_*H*_4_*PH*_3_*P*_2_*H*_2_*P*_2_*HPH*_2_*PHPH*_2_*P*_2_*H*_2_*P*_3_*HP*_8_*H*_2_
S2-2	48	-34	*H*_4_*PH*_2_*PH*_5_*P*_2_*HP*_2_*H*_2_*P*_2_*HP*_6_*HP*_2_*HP*_3_*HP*_2_*H*_2_*P*_2_*H*_3_*PH*
S2-3	48	-34	*PHPH*_2_*PH*_6_*P*_2_*HPHP*_2_*HPH*_2_(*PH*)_2_*P*_3_*H*(*P*_2_*H*_2_)_2_*P*_2_*H*_*P*_*HP*_2_*HP*
S2-4	48	-33	*PHPH*_2_*P*_2_*HPH*_3_*P*_2_*H*_2_*PH*_2_*P*_3_*H*_5_*P*_2_*HPH*_2_(*PH*)_2_*P*_4_*HP*_2_(*HP*)_2_
S2-5	48	-32	*P*_2_*HP*_3_*HPH*_4_*P*_2_*H*_4_*PH*_2_*PH*_3_*P*_2_(*HP*)_2_*HP*_2_*HP*_6_*H*_2_*PH*_2_*PH*
S2-6	48	-32	*H*_3_*P*_3_*H*_2_*PH*(*PH*_2_)_3_*PHP*_7_*HPHP*_2_*HP*_3_*HP*_2_*H*_6_*PH*
S2-7	48	-32	*PHP*_4_*HPH*_3_*PHPH*_4_*PH*_2_*PH*_2_*P*_3_*HPHP*_3_*H*_3_(*P*_2_*H*_2_)_2_*P*_3_*H*
S2-8	48	-31	*PH*_2_*PH*_3_*PH*_4_*P*_2_*H*_3_*P*_6_*HPH*_2_*P*_2_*H*_2_*PHP*_3_*H*_2_(*PH*)_2_*PH*_2_*P*_3_
S2-9	48	-34	(*PH*)_2_*P*_4_(*HP*)_2_*HP*_2_*HPH*_6_*P*_2_*H*_3_*PHP*_2_*HPH*_2_*P*_2_*HPH*_3_P_4_*H*
S2-10	48	-33	*PH*_2_*P*_6_*H*_2_*P*_3_*H*_3_*PHP*_2_*HPH*_2_(*P*_2_*H*)_2_*P*_2_*H*_2_*P*_2_*H*_7_*P*_2_*H*_2_

**Table 2:** Performance comparison of various algorithms for the 2D HP Protein Folding Problem. Comparison of the solution quality obtained in 2D by the evolutionary algorithm of Unger and Moult (EA) [14], the evolutionary Monte Carlo algorithm of Liang and Wong (EMC) [19], the multi-self-overlap ensemble algorithm of Chickenji *et al. *(MSOE) [17], the pruned-enriched Rosenbluth method (PERM) and ACO-HPPFP-3. For EA and EMC, the reported energy values are the lowest among five independent runs, and the values in parentheses are the numbers of valid conformations scanned before the lowest energy values were found. Missing entries indicate cases where the respective method has not been tested on a given instance. The CPU times reported in parentheses for MSOE have been determined on a 500 MHz CPU, and those for PERM and ACO-HPPFP-3 are based on 100 – 200 runs per instance on our reference 2.4 GHz Pentium IV machine. The energy values shown in bold face correspond to currently best known solution qualities.

ID	*E*	GA	EMC	MSOE	PERM *t*_1_	PERM *t*_2_	PERM *t*_*exp*_	ACO
S1-1	**-9**	**-9 **(30 492)	**-9 **(9 374)		**-9 **(< 1 sec)	**-9 **(< 1 sec)	**-9 **(< 1 sec)	**-9 **(< 1 sec)
S1-2	**-9**	**-9 **(30 491)	**-9 **(6 929)		**-9 **(< 1 sec)	**-9 **(< 1 sec)	**-9 **(< 1 sec)	**-9 **(< 1 sec)
S1-3	**-8**	**-8 **(20 400)	**-8 **(7 202)		**-8 **(6 sec)	**-8 **(< 1 sec)	**-8 **(2 sec)	**-8 **(< 1 sec)
S1-4	**-14**	**-14 **(301 339)	**-14 **(12 447)		**-14 **(< 1 sec)	**-14 **(< 1 sec)	**-14 **(< 1 sec)	**-14 **(4 sec)
S1-5	**-23**	**-23 **(126 547)	**-23 **(165 791)		**-23 **(3 min)	**-23 **(< 1 sec)	**-23 **(2 sec)	**-23 **(1 min)
S1-6	**-21**	**-21 **(592 887)	**-21 **(74 613)		**-21 **(3 sec)	**-21 **(3 sec)	**-21 **(3 sec)	**-21 **(15 sec)
S1-7	**-36**	-34 (208 781)	-35 (203 729)		**-36 **(7 sec)	**-36 **(3 sec)	**-36 **(4 sec)	**-36 **(20 min)
S1-8	**-42**	-37 (187 393)	-39 (564 809)	-39	-42 (78 hrs)	-42 (78 hrs)	-42 (78 hrs)	**-42 **(1.5 hrs)
S1-9	**-53**		-52 (44 029)		**-53 **(64 sec)	**-53 **(60 sec)	**-53 **(1 min)	**-53 **(20% of runs 1 days)
S1-10	**-50**			**-50 **(50 hrs)	**-50 **(50% of runs 1 hrs)	**-50 **(20 min)	**-50**	-49 (12 hrs)
S1-11	**-48**			-47	**-48 **(9 min)	**-48 **(7 min)	**-48 **(8 min)	-47 (10 hrs)

**Table 3:** Performance comparison of various algorithms for the 3D HP Protein Folding Problem. Comparison of the solution quality obtained in 3D by the hydrophobic zipper (HZ) algorithm [23], the constraint-based hydrophobic core construction method (CHCC) [26], the core-directed chain growth algorithm (CG) [25], the contact interactions (CI) algorithm [24], the pruned-enriched Rosenbluth method (PERM) and ACO-HPPFP-3. For CI, only the best energies obtained are shown. For HZ, CHCC and CG, the reported CPU times are taken from [25]; these are the expected times for finding optimal solutions on a Sparc 1 workstation. In the case of HZ, the reported CPU times are based on an extrapolation from the measured times required for finding suboptimal conformations with the energy values listed here. The CPU times for PERM and ACO-HPPFP-3 were determined on our reference 2.4 GHz Pentium IV machine based on 50 – 100 runs per instance. The energy values shown in bold face correspond to currently best known solution qualities.

ID	*E*	HZ	CHCC	CG	CI	PERM *t*_1_	PERM *t*_2_	PERM *t*_*exp*_	ACO
S2-1	**-32**	-31(4 hrs)	**-32 **(30 min)	**-32 **(9.4 min)	**-32**	**-32 **(0.1 min)	**-32 **(0.5 min)	**-32 **(0.2 min)	**-32 **(30 min)
S2-2	**-34**	-32 (18 hrs)	**-34 **(2.3 min)	**-34 **(35 min)	-33	**-34 **(0.3 min)	**-34 **(48 min)	**-34 **(0.6 min)	**-34 **(420 min)
S2-3	**-34**	-31 (23 hrs)	**-34 **(30 min)	**-34 **(62 min)	-32	**-34 **(0.1 min)	**-34 **(4 days)	**-34 **(0.2 min)	**-34 **(120 min)
S2-4	**-33**	-30 (19 days)	**-33 **(71 min)	**-33 **(29 min)	-32	**-33 **(2 min)	**-33 **(4 min)	**-33 **(3 min)	**-33 **(300 min)
S2-5	**-32**	-30 (1.3 days)	**-32 **(32 min)	**-32 **(12 min)	**-32**	**-32 **(0.5 min)	**-32 **(19 min)	**-32 **(1 min)	**-32 **(15 min)
S2-6	**-32**	-29 (2.1 days)	**-32 **(80 min)	**-32 **(460 min)	-30	**-32 **(0.5 min)	**-32 **(0.1 min)	**-32 **(0.2 min)	**-32 **(720 min)
S2-7	**-32**	-29 (2.5 days)	**-32 **(110 min)	**-32 **(64 min)	-30	**-32 **(0.5 min)	**-32 **(2 days)	**-32 **(1 min)	**-32 **(720 min)
S2-8	**-31**	-29 (4 hrs)	**-31 **(530 min)	**-31 **(38 min)	-30	**-31 **(0.3 min)	**-31 **(8 min)	**-31 **(0.6 min)	**-31 **(120 min)
S2-9	**-34**	-31(4.5 hrs)	**-34 **(8.3 min)	-33	-32	**-34 **(5 min)	**-34 **(10 min)	**-34 **(7 min)	**-34 **(450 min)
S2-10	**-33**	**-33 **(1.1 hr)	**-33 **(4.8 min)	**-33**(1.1 min)	-32	**-33 **(0.01 min)	**-33 **(0.01 min)	**-33 **(0.01 min)	**-33 **(60 min)

## References

[B1] Dorigo M, Maniezzo V, Colorni A (1991). Positive feedback as a search strategy. Tech rep, 91-016, Dip Elettronica, Politecnico di Milano, Italy.

[B2] Dorigo M, Maniezzo V, Colorni A (1996). The Ant System: Optimization by a colony of cooperating agents. IEEE Transactions on Systems, Man, and Cybernetics-Part B.

[B3] Dorigo M, Di Caro G, Corne D, Dorigo M, Glover F (1999). New Ideas in Optimization. New Ideas in Optimization.

[B4] Dorigo M, Di Caro G, Gambardella LM (1999). Ant Algorithms for Discrete Optimization. Artificial Life.

[B5] Dorigo M, Stützle T (2004). Ant Colony Optimization.

[B6] Shmygelska A, Hernandez R, Hoos HH (2002). An Ant Colony Optimization Algorithm for the 2D HP Protein Folding Problem. Proc of the 3rd Intl Workshop on Ant Algorithms, ANTS LNCS 2463.

[B7] Shmygelska A, Hoos HH (2003). An Improved Ant Colony Optimisation Algorithm for the 2D HP Protein Folding Problem. Proc of the 16th Canadian Conference on Artificial Intelligence, LNCS 2671.

[B8] Unger R, Moult J (1993). Finding the lowest Free-Energy Conformation of a protein is an NP-hard problem – Proof and Implications. Bull Math Biol.

[B9] Lau KF, Dill KA (1989). lattice statistical mechanics model of the conformation and sequence space of proteins. Macromolecules.

[B10] Richards FM (1977). Areas, volumes, packing, and protein structures. Annu Rev Biophys Bioeng.

[B11] Krasnogor N, Pelta D, Lopez PM, Mocciola P, de la Canal E, Alpaydin C (1998). Genetic algorithms for the protein folding problem: a critical view. Proc of Engineering of Intelligent Systems.

[B12] Krasnogor N, Hart WE, Smith J, Pelta DA (1999). Protein structure prediction with evolutionary algorithms. Proc of the Genetic and Evolutionary Computation conference.

[B13] Patton AWP, Goldman E (1995). A standard GA approach to native protein conformation prediction. Proc of the 6th Intl Conf Genetic Algorithms.

[B14] Unger R, Moult J (1993). Genetic algorithms for protein folding simulations. J of Mol Biol.

[B15] Unger R, Moult J (1993). A genetic algorithm for three dimensional protein folding simulations. Proc of the 5th Intl Conf on Genetic Algorithms.

[B16] Bastolla U, Fravenkron H, Gestner E, Grassberger P, Nadler W (1998). Testing a New Monte Carlo algorithm for the protein folding problem. Proteins.

[B17] Chikenji G, Kikuchi M, Iba Y (1999). Multi-Self-Overlap Ensemble for protein folding: ground state search and thermodynamics. Condensed Materials Archive.

[B18] Hsu HP, Mehra V, Nadler W, Grassberger P (2003). Growth Algorithm for Lattice Heteropolymers at Low Temperatures. J Chem Phys.

[B19] Liang F, Wong WH (2001). Evolutionary Monte Carlo for protein folding simulations. J Chem Phys.

[B20] O'Toole EM, Panagiotopoulos AZ (1992). Monte Carlo simulation of folding transitions of simple model proteins using a chain growth algorithm. J Chem Phys.

[B21] Ramakrishnan R, Ramachandran B, Pekny JF (1997). A dynamic Monte Carlo algorithm for exploration of dense conformational spaces in heteropolymers. J Chem Phys.

[B22] Sali A, Shakhnovich E, Karplus M (1994). How does a protein fold?. Nature.

[B23] Dill KA, Fiebig KM, Chan HS (1993). Cooperativity in Protein-Folding Kinetics. Proc Natl Acad Sci USA.

[B24] Toma L, Toma S (1996). Contact interactions method: A new algorithm for protein folding simulations. Protein Sci.

[B25] Beutler T, Dill K (1996). A fast conformational search strategy for finding low energy structures of model proteins. Protein Sci.

[B26] Yue K, Dill KA (1995). Forces of Tertiary Structural Organization in Globular Proteins. Proc Natl Acad Sci USA.

[B27] Backofen R, Will S (2003). A Constraint-Based Approach to Structure Prediction for Simplified Protein Models that Outperforms Other Existing Methods. Proc XIX Intl Conf on Logic Programming.

[B28] Torrie GM, Valleau JP (1977). Nonphysical sampling distributions in MC free energy estimation: Umbrella sampling. J Comput Phys.

[B29] Gront D, Kolinski A, Skolnick J (2000). Comparison of three Monte Carlo conformational search strategies for a proteinlike homopolymer model: Folding thermodynamics and identification of low-energy structures. J Chem Phys.

[B30] Mitsutake A, Sugita Y, Okamoto Y (2003). Replica-exchange multicanonical and multicanonical replica-exchange Monte Carlo simulations of peptides. I. Formulation and benchmark test. J Chem Phys.

[B31] Berg BA, Neuhaus T (1992). Multicanonical ensemble: A new approach to simulate first-order phase transitions. Phys Rev Lett.

[B32] Irbäck A, Grassberger P, Barkema GT, Nadler W, (1998). Dynamic-parameter algorithms for protein folding. Monte Carlo Approach to Biopolymers and Protein Folding.

[B33] Backofen R, Will S, Clote P (2000). Algorithmic approach to quantifying the hydrophobic force contribution in protein folding. Proc of the 5th Pacific Symposium on Biocomputing.

[B34] Hsu HP, Mehra V, Nadler W, Grassberger P (2003). Growth-based Optimisation Algorithm for Lattice Heteropolymers. Phys Rev E.

[B35] Nandi T, B-Rao C, Ramachandran S (2002). Comparative Genomics using Data Mining tools. J Bioscience.

[B36] Sayle R, Milner-White EJ (1995). RASMOL – Biomolecular Graphics for All. Trends Biochem Sci.

[B37] Creighton TE (1992). Protein Folding.

[B38] Plaxco KW, Simons KT, Baker D (1998). Contact order, transition state placement and the refolding rates of single domainproteins. J Mol Biol.

[B39] Hoos HH, Stützle T (1998). On the empirical evaluation of Las Vegas algorithms. Proc of the 14th Conference on Uncertainty in Artificial Intelligence.

[B40] Sali A, Shakhnovich E, Karplus M (1994). Kinetics of protein folding – A lattice model study of the requirements for folding tothe native state. J Mol Biol.

[B41] Stützle T, Hoos HH (2000). MAX-MIN Ant System. Future Generation Computer Systems.

[B42] Hoos HH, Stützle T (2004). Stochastic Local Search: Foundations and Applications.

[B43] Parkes A, Walser JP (1996). Tuning Local Search for Satisfiability Testing. Proc of the Applications of Artificial Intelligence Conf.

[B44] HP Benchmarks. http://www.cs.sandia.gov/tech_reports/compbio/tortilla-hp-benchmarks.html.

[B45] Konig R, Dandekar T (1999). Improving Genetic Algorithms for Protein Folding simulations by systematic crossover. Biosystems.

