# Supplementary material for: An ant colony optimisation algorithm for the 2D and 3D hydrophobic polar protein folding problem
Source: BMC Bioinformatics. 2005 Feb 14;6:30. doi: 10.1186/1471-2105-6-30 (PMC555464; doi:10.1186/1471-2105-6-30)
Supplement: Additional File 1 — Additional information on biological and randomly generated HP sequences. This file (in .pdf format) contains tables providing additional information on our new test sets of biological and randomly generated HP sequences and the results from our computational experiment with ACO and PERM. [file 1471-2105-6-30-S1.pdf]

Supplementary Material

Additional File 1

The following tables provide additional information on our new test sets of biological and randomly generated HP sequences and the results from our computational experiment with ACO-HPPFP-3 and PERM. For the biological sequences, we show the PDB IDs of the original protein sequences. Note that in our translation from protein sequences into HP strings, non-standard amino acid symbols, such as X and Z, were skipped; consequently, some of our HP strings differ in length from the respective PDBSELECT sequences.

ID	PDB ID	HP sequence	Length
B30-1	1HA9:A	phpphhhphphhppppppppphhhphpphphhph	34
B30-2	1CE4:A	ppphppppppphphhhphphhhpphhhhphpphpp	35
B30-3	1FWO:A	pphpppphpphhhppppphhhhpphphhhhpphhp	35
B30-4	1ZTA	hpphpphppphpphhppphphppphhphpphhhpp	35
B30-5	1G1Z:A	ppphphhhpphhhhpphhpphhphhhphphp	31
B30-6	1BZG	hhpppphhppphpphpphppphhhpphhhphpph	34
B30-7	1BH7	hphhpphhhhhphhphphphhhhpphpphphph	33
B30-8	1B4G	hhpphphphphppphpphhpppphppphh	29
B30-9	1BNX:A	phhhhhpphpphhhhhhhhhphhhphhhhhhpp	33
B30-10	1FCT	hhhhhppphhhphhhphhhphhphhpphpphh	32

Table A-1: Biological sequences of length ≈ 30

ID	E_{min} (2D)	PERM t_1	PERM t_2	PERM t_{exp}	ACO t_{avg}
B30-1	-12	0.43	0.56	0.49	0.71
B30-2	-12	0.15	4.09	0.29	1.08
B30-3	-14	0.15	0.02	0.04	1.19
B30-4	-10	2.5	0.3	0.54	3.64
B30-5	-13	0.10	0.17	0.12	0.22
B30-6	-13	0.84	10.40	1.55	70.94
B30-7	-16	0.02	0.02	0.02	0.19
B30-8	-8	0.09	0.003	0.006	0.17
B30-9	-18	0.002	0.008	0.003	0.06
B30-10	-16	0.003	12.50	0.006	4.08

Table A-2: Performance comparison of PERM and ACO-HPPFP-3 on biological sequences of length ≈ 30 in 2D

ID	E_{min} (3D)	PERM t_1	PERM t_2	PERM t_{exp}	ACO t_{avg}
B30-1	-17	1.14	1.03	1.08	1.19
B30-2	-16	1.06	1.3	1.17	2.29
B30-3	-18	1.20	1.20	1.20	2.34
B30-4	-16	3.4	1.2	1.77	20.18
B30-5	-20	2.2	3.04	2.55	65.71
B30-6	-19	1.20	1.50	1.33	23.18
B30-7	-25	1.10	2.40	1.51	62.36
B30-8	-13	1.10	1.07	1.09	1.74
B30-9	-26	1.20	1.09	1.14	1.57
B30-10	-24	1.8	3 291	3.60	163.23

Table A-3: Performance comparison of PERM and ACO-HPPFP-3 on biological sequences of length ≈ 30 in 3D

ID	PDB ID	HP sequence	Length
B50-1	1KBF:A	hphppphppphhpphpphpppphhhhhppppphpppppppppphhhpp	49
B50-2	1KBH:A	phppppphhhpphpphhpppphphhpphpphhhhhphhpphphhphp	47
B50-3	1G9P:A	hhhphhhphppppppppppphphhppphpphpphhhhhhhhhhhhh	45
B50-4	1YUJ:A	hphpphpphhhppphppppppppphhpphhphhhhppppphppphphpphh	50
B50-5	1GAB	phpphhhpphppphhhphpphhhppphhhphhpphpphpphphhppphhphph	53
B50-6	2BRZ	pppppphhpphhhppppphhppppphpppphppphpphpphhppppphpphpphpph	53
B50-7	1VPC	pphphhphhhphhpphhhhphphhppppphhhhpppppppphhppp	45
B50-8	1VIB	hphphhhhhhppppppppphphphppphphhhpphpphhpphhppppppphppppp	54
B50-9	1CEU:A	hpphhppphhppphhpphphphhpphppphhpphhphhhpphhpphhpphh	51
B50-10	1CFH	hpphphpphhphphppppphppppphpphpphhppppppppphhpphhp	47

Table A-4: Biological sequences of length ≈ 50

ID	E_{min} (2D)	PERM			ACO
		t_1	t_2	t_{exp}	t_{avg}
B50-1	-12	5.60	3.90	4.60	39.5
B50-2	-19	650.40	0.90	1.80	283.31
B50-3	-20	3.80	0.04	0.08	2.47
B50-4	-17	3.33	2.24	2.68	184.5
B50-5	-22	4.90	117.80	9.41	820.37
B50-6	-14	0.43	0.74	0.55	2.08
B50-7	-17	270.70	298.80	284.06	130.35
B50-8	-14	0.04	2.16	0.07	0.29
B50-9	-21	0.40	0.23	0.29	55.32
B50 -10	-14	0.27	0.3	0.28	3.22

Table A-5: Performance comparison of PERM and ACO-HPPFP-3 on biological sequences of length ≈ 50 in 2D

ID	E_{min} (3D)	PERM			ACO
		t_1	t_2	t_{exp}	t_{avg}
B50-1	-18	2.08	1.50	1.74	14.65
B50-2	-29	43.60	18.20	25.68	701.84
B50-3	-28	1.40	1.30	1.35	5.52
B50-4	-28	2.29	1 822.2	117.90	(-27)
B50-5	-37	18.2	1 235.9	164.87	(-36)
B50-6	-25	2.80	10.7	4.44	1 638.29
B50-7	-26	406.90	2.70	5.36	814.11
B50-8	-25	10.05	(-24)	-	1026.12
B50-9	-36	3 492.6	4.03	16.10	(-35)
B50 -10	-22	2.50	1.40	1.80	200.68

Table A-6: Performance comparison of PERM and ACO-HPPFP-3 on biological sequences of length ≈ 50 in 3D

ID	HP sequence	Length	H fraction
R30-1	pphphpphpphphppppphphhpphhhpph	30	0.40
R30-2	hpppppphhphhhpphppphhhhhhhhpph	30	0.53
R30-3	pphpphphppphhppppphhpphphphhhhh	30	0.47
R30-4	pphhhhpppppphphhpphphpppppphphh	30	0.40
R30-5	hhppphhphpphhphhhpphphpphphpph	30	0.50
R30-6	phhpphphppppphhhphpppphppphhhh	30	0.43
R30-7	phhhphhhppppphhphphphhhpppppph	30	0.47
R30-8	ppphpphhphphhpphpphphhhphhphph	30	0.50
R30-9	hhphhppppphppphphpphpphpphhphph	30	0.43
R30-10	hphphpphpphphhppphphhhhphhphhp	30	0.53

Table A-7: Random sequences of length 30

ID	E_{min} (2D)	PERM t_1	t_2	t_{exp}	ACO t_{avg}
R30-1	-90	0.4	0.001	0.002	0.27
R30-2	-13	1.60	0.011	0.022	0.58
R30-3	-10	0.14	0.001	0.002	0.17
R30-4	-9	0.04	0.07	0.05	0.26
R30-5	-13	0.01	0.038	0.02	0.44
R30-6	-11	86.3	0.33	0.65	12.85
R30-7	-8	0.007	0.03	0.01	0.10
R30-8	-12	0.033	0.017	0.045	0.38
R30-9	-9	0.002	0.11	0.004	0.14
R30-10	-12	0.007	0.001	0.002	0.34

Table A-8: Performance comparison of PERM and ACO-HPPFP-3 on random sequences of length 30 in 2D

ID	E_{min} (3D)	PERM t_1	t_2	t_{exp}	ACO t_{avg}
R30-1	-13	0.16	0.16	0.16	0.47
R30-2	-18	0.22	0.44	0.29	3.44
R30-3	-15	1.00	1.85	1.30	17.81
R30-4	-12	0.14	0.26	0.18	0.25
R30-5	-19	0.53	0.51	0.52	28.77
R30-6	-14	0.22	0.10	0.14	1.11
R30-7	-11	0.118	0.067	0.09	0.055
R30-8	-17	0.19	0.28	0.225	0.57
R30-9	-14	0.22	0.24	0.23	1.48
R30-10	-19	2.22	0.27	0.48	16.69

Table A-9: Performance comparison of PERM and ACO-HPPFP-3 on random sequences of length 30 in 3D

ID	HP sequence	Length	H fraction
R50-1	ppphphphppppphphhhphhhpppphphphhppphphphhhhphpphhh	50	0.48
R50-2	hhhhhhphppphppppphhhpphhhhhphpphhhhpphppphppphhppph	50	0.52
R50-3	hhhphhphphhppphpphhhphhhhhhhphppphphhhphppphphpppp	50	0.54
R50-4	hhhhpphpphhpphhhppppppphpphhppppphhhhphhhpppphppphhh	50	0.48
R50-5	ppphhppphpphphpphhphphhhhhhhhpphhhpphppppphpppphphp	50	0.46
R50-6	pphpphphphhhpphpppppphpphhppppphphhhhphhphhhhpphhph	50	0.48
R50-7	phhppphphpphhppppphhhphphhhphhphhphphpphhhphphhhhhp	50	0.56
R50-8	hppppphphphpphphpphhhhhhpphhhhhphppphhpphphhpppphhp	50	0.50
R50-9	pphphhphhphpphppppphphphphphpphhppppppphpphhhhhpphhh	50	0.46
R50-10	hhphphppppphpphphpppppphphppphpppppphhhphhhhhphhphph	50	0.44

Table A-10: Random sequences of length 50

ID	E_{min} (2D)	PERM t_1	t_2	t_{exp}	ACO t_{avg}
R50-1	-29	575.90	1.05	2.09	367.70
R50-2	-34	1.40	2.40	1.77	1 150.10
R50-3	-32	1.00	5.50	1.69	893.66
R50-4	-32	2.70	2.40	2.54	1 329.01
R50-5	-32	(-31)	5.49	-	(-31)
R50-6	-32	3.8	1.6	2.25	1 153.81
R50-7	-38	15 322.20	46.20	92.12	(-37)
R50-8	-33	1.30	0.91	1.07	837.13
R50-9	-30	9892.00	1.70	3.40	1000.49
R50-10	-27	72.80	1.80	3.51	530.84

Table A-11: Performance comparison of PERM and ACO-HPPFP-3 on random sequences of length 50 in 2D

ID	E_{min} (3D)	PERM t_1	t_2	t_{exp}	ACO t_{avg}
R50-1	-29	575.90	1.05	2.09	367.70
R50-2	-34	1.40	2.40	1.77	1 150.10
R50-3	-32	1.00	5.50	1.69	893.66
R50-4	-32	2.70	2.40	2.54	1 329.01
R50-5	-32	(-31)	5.49	-	(-31)
R50-6	-32	3.8	1.6	2.25	1 153.81
R50-7	-38	15 322.20	46.20	92.12	(-37)
R50-8	-33	1.30	0.91	1.07	837.13
R50-9	-30	9892.00	1.70	3.40	1000.49
R50-10	-27	72.80	1.80	3.51	530.84

Table A-12: Performance comparison of PERM and ACO-HPPFP-3 on random sequences of length 50 in 3D
